# Epigenetic Aging in Brain Tissue of the Self‐Fertilizing Vertebrate, *Kryptolebias marmoratus*

**DOI:** 10.1002/ece3.73881

**Published:** 2026-06-21

**Authors:** Justine Bélik, Frédéric Silvestre

**Affiliations:** ^1^ Laboratory of Evolutionary and Adaptive Physiology, Institute of Life, Earth and Environment University of Namur Namur Belgium

**Keywords:** brain aging, DNA methylation, epigenetic clock, mangrove rivulus

## Abstract

DNA methylation changes predictably with age across taxa, but in most species, these patterns are confounded by genetic variation. As a result, age‐predictive methylation models have mostly been developed in genetically heterogeneous, cross‐fertilizing organisms, limiting inference about epigenetic aging per se. Disentangling epigenetic and genetic effects is therefore essential for understanding aging, adaptation, and evolution. Here, we exploit the mangrove rivulus (*Kryptolebias marmoratus*), one of only two known self‐fertilizing vertebrates (together with *K. hermaphroditus*), to examine epigenetic aging in a system of naturally occurring near‐isogenic individuals. Using reduced‐representation bisulfite sequencing of 89 brain samples spanning 60–1100 days of age, we identified 40 CpG sites whose methylation levels predict chronological age with high accuracy (*R* ^2^ > 0.96, Median Absolute Error of 28.7 days). These 40 age‐associated CpG sites were linked to nearby genes with known roles in cellular maintenance and neurodegeneration. These include genes implicated in aging and neurodegenerative processes across vertebrates, such as lamin‐A, the aryl hydrocarbon receptor, and genes associated with Alzheimer's disease in humans. By leveraging a self‐fertilizing vertebrate, this study demonstrates that DNA methylation undergoes consistent, age‐associated changes across the lifespan in the near absence of genetic variation. Our results establish self‐fertilizing vertebrates as powerful models for disentangling epigenetic aging from genetic effects and provide a foundation for comparative and evolutionary studies of aging.

## Graphical abstract

Using the self‐fertilizing vertebrate *Kryptolebias marmoratus*, we developed an epigenetic clock from brain tissue that predicts chronological age with high accuracy, demonstrating that DNA methylation changes consistently with age even in the near absence of genetic variation. The 40 age‐associated CpG sites identified map to genes with conserved roles in cellular maintenance, neurodegeneration and stress response. This work establishes self‐fertilizing vertebrates as uniquely powerful models for disentangling epigenetic aging from genetic effects, with broad implications for comparative and evolutionary aging research.

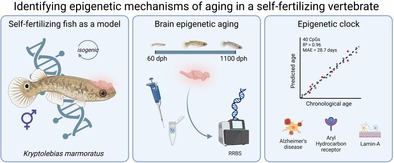

## 1 Introduction

Aging is a progressive, time‐dependent decline in physiological integrity, leading to impaired function and an increased risk of death among most living organisms (Jones et al. 2015; López‐Otín et al. 2013). It is considered the primary risk factor for several major human pathologies, including cancer, diabetes, cardiovascular disorders, and neurodegenerative diseases, highlighting its central role in ongoing scientific research. López‐Otín et al. (2013) initially described nine “Hallmarks of Aging”: genomic instability, telomere attrition, epigenetic alterations, loss of proteostasis, deregulated nutrient sensing, mitochondrial dysfunction, cellular senescence, stem cell exhaustion, and altered intercellular communication. These hallmarks have been widely studied and validated. However, the list has since been updated to include disabled macroautophagy, chronic inflammation, and dysbiosis (López‐Otín et al. 2023). Each hallmark represents a distinct yet interconnected entry point for investigating the biology of aging, as they collectively influence and reinforce one another.

Among the hallmarks of aging, epigenetic alterations play a significant role. These include changes in DNA methylation patterns, abnormal posttranslational modifications of histones, aberrant chromatin remodeling, and deregulated functions of noncoding RNAs (López‐Otín et al. 2023). Such modifications can profoundly impact gene expression, contributing to the onset and progression of several age‐related pathologies. In particular, the age‐associated accumulation of DNA methylation (DNAm) modifications has been extensively studied (Seale et al. 2022). DNAm involves the addition of a methyl group to a cytosine, most commonly at CpG dinucleotides in vertebrates (i.e., a cytosine located 5′ of a guanine). This process is achieved by a set of enzymes from the DNA methyltransferase (DNMT) family, notably, DNMT1 (which copies preexisting DNA methylation motifs onto the new strand during mitosis) and DNMT3a/3b (which perform *de novo* methylation). DNAm can be reversed through two primary processes: active demethylation, which is mediated by ten‐eleven translocases (TETs) dioxygenases, and passive demethylation, which occurs when functional DNMT activity is absent during DNA replication (Davletgildeeva and Kuznetsov 2024).

Current literature demonstrates that aging is strongly correlated with DNAm, with substantial functional consequences. Early studies suggested a global hypomethylation with aging (Fuke et al. 2004; Singhal et al. 1987; Wilson et al. 1987) but more recent studies produced conflicting results and do not support this hypothesis (Fasolino et al. 2017; Lister et al. 2013; Raddatz et al. 2013). Either way, DNAm is both gained and lost with aging, resulting in a more variable DNAm pattern in older individuals (Meyer and Schumacher 2024). This includes hypermethylation of previously unmethylated regions and hypomethylation of previously methylated regions, increasing the overall entropy of the DNAm pattern over time (Meyer and Schumacher 2024; Tarkhov et al. 2024; Tong et al. 2024). However, some CpG sites exhibit a strong, consistent correlation with chronological age, emphasizing their potential as age predictors (De Paoli‐Iseppi et al. 2017). These alterations are often tissue‐ and species‐specific (Unnikrishnan et al. 2018). These so‐called “clock CpGs” form the basis of epigenetic clocks, sets of carefully selected loci across the genome that are collectively capable of predicting chronological age with high accuracy (Piferrer and Anastasiadi 2023).

Key epigenetic clocks include the model developed by Horvath (2013), constructed from 8000 human multi‐tissue samples, which uses 353 CpGs to estimate age with a median error of 3.6 years. The Hannum clock (Hannum et al. 2013), which is based solely on human blood samples, highlights specific components of human aging. Levine et al. (2018) advanced the concept of biological age versus chronological age by incorporating clinical health biomarkers into the clock's design. In parallel, numerous epigenetic clocks have been built across the tree of life. In mammals, examples include *Mus musculus* (Spiers et al. 2016; Stubbs et al. 2017) and *Canis lupus familiaris* (Thompson et al. 2017). In birds, clocks have been constructed for *Gallus gallus* (Gryzinska et al. 2013) and *Coturnix japonica* (Andraszek et al. 2014). Clocks also exist for reptiles, such as *Alligator mississippiens* (Nilsen et al. 2016), elasmobranchs with *Stegostoma tigrinum* (Bock et al. 2026) and fish, including the European seabass (Piferrer et al. 2024) and the haddock (Strand et al. 2025). In addition, the first epigenetic clocks for invertebrate species are now emerging (Brink et al. 2024; Guynes et al. 2024). The recent development of a universal pan‐mammalian clock suggests that epigenetic aging is evolutionarily conserved across mammals (Lu et al. 2023). Similarly, a study on cetaceans introduced a multi‐species, two‐tissue clock, highlighting the potential to develop tools applicable across diverse taxa (Zoller et al. 2025).

The further development of epigenetic clocks is valuable, as it offers insights into aging mechanisms and related diseases. Comparative studies can identify universal and species‐specific aspects of aging and epigenetic regulation (Bertucci‐Richter and Parrott 2023). Moreover, several independent studies have demonstrated that epigenetic age is indicative of past and/or present health status (Caulton et al. 2022). Epigenetic clocks offer yet underexploited potential in the context of breeding programs and fisheries management (Piferrer and Anastasiadi 2023), as well as conservation work (Heydenrych et al. 2021). Age estimation notably enables the calculation of growth rates (to assess species productivity and growth variation over time), mortality rates (natural and fishing mortality rates), and population age structure (Piferrer and Anastasiadi 2023).

Most epigenetic clocks are based on easily accessible tissues, such as blood or caudal fins, to enable nonlethal and minimally invasive age estimation (Piferrer et al. 2024; Piferrer and Anastasiadi 2023). However, these tissues may not be the most relevant for increasing our fundamental understanding of aging. In contrast, investigating the aging brain methylome is critically important, given its influence on neural function and the development of neurodegenerative diseases (Liu et al. 2009; Pellegrini et al. 2021; Thrush et al. 2022; Yang et al. 2015). Only a few epigenetic clocks have been constructed via postmortem brain samples, either in nonhuman primates (Horvath et al. 2021; Jasinska et al. 2021) or humans (Grodstein et al. 2021; Shastri et al. 2024). To our knowledge, only two studies have focused on the brain in the context of the epigenetic clock (Coninx et al. 2020; Stubbs et al. 2017). Thus, expanding our knowledge of brain‐specific methylation patterns in the context of aging has become an essential objective for both epigenetic and neuroscience research.

Here, we focus on the mangrove rivulus, *Kryptolebias marmoratus*. With its sister species, *Kryptolebias hermaphoditus*, they are the only known self‐fertilizing vertebrates (Costa et al. 2010). This unique feature allows us to study naturally clonal lineage and disentangle epigenetic and genetic effects. *K. marmoratus* belongs to a broad group of killifish species and is distributed along the coasts of Florida to northern Brazil, closely following the red mangrove *Rhizophora mangle* (Taylor 2012). Its reproduction strategy is remarkable, as it involves a rare mixed mating system known as androdioecy (Costa et al. 2010). Populations of *K. marmoratus* are composed of self‐fertilizing hermaphrodites, which produce clonal offspring after a few generations, and males, which can cross‐fertilize unfertilized eggs laid by hermaphrodites. In natural populations, there is a gradient in male frequency, resulting in varying levels of genetic diversity, with selfing rates ranging from 49% to 97% (Chapelle 2023). The population used in our study originates from Emerson Point Preserve, Florida, and is characterized by an extremely high selfing rate of 97% and an observed heterozygosity of zero. The virtual absence of genetic variation offers a unique opportunity to study epigenetic variation without the confounding effects of underlying genetic differences, and is essential to understand aging, adaptation and evolution.

We analyzed 989 brain samples from hermaphroditic *Kryptolebias marmoratus* individuals of known age and spanning the species' lifespan (60 days to 3.5 years post‐hatching). Using reduced‐representation bisulfite sequencing (RRBS), we identified 40 CpG sites whose combined methylation profiles accurately predict chronological age (Median Absolute Error, 28.7 days; *R* ^2^ = 0.96). Genomic annotations of these sites revealed associations with nearby genes involved in aging‐related processes, including DNA damage repair, mTOR signaling, inflammation, and autophagy, as well as genes implicated in Alzheimer's disease (AD) in human studies. Together, these results demonstrate that age‐associated DNA methylation changes arise consistently across the lifespan of a near‐isogenic vertebrate, providing evidence that epigenetic aging dynamics can be detected largely independently of genetic variation.

## 2 Material and Methods

### 2.1 Mangrove Rivulus Aging Colony

*Kryptolebias marmoratus* individuals were collected in 2019 from Emerson Point Preserve, Florida (N25°01′ 45.64″, W80°29′ 49.24″, permit number SAL‐24‐1132**A**‐SR) and used to establish the F0 generation at the University of Namur. The fish were individually housed in 500 mL plastic containers filled with 300 mL of 12 ± 1 parts per thousand (ppt) saltwater (Instant Ocean Sea salt) and maintained in a climate‐controlled room at 25°C ± 1°C under a 12:12 light:dark photoperiod. The fish were fed daily *ad libitum* with live *Artemia salina* .

For the epigenetic clock analysis, we selected 96 hermaphrodite individuals, covering the major life span of the mangrove rivulus (from 60 to 1100 days). The age of the individuals is known to the day, as they hatched in the laboratory. The number of fish was decided following (Mayne et al. 2021), knowing that our species is self‐fertilizing, which reduces genetic and therefore epigenetic variability, and that we use only one sex. The fish were between the 2nd and 6th generations derived from the F0 stock (field individuals). Out of the 96 fish selected, four were excluded after being identified as secondary males, and two were removed because of insufficient DNA yield. Once they reached the expected age, the fish were euthanized by rapid cooling (immersion in water between 0°C and 4°C for 20 s), and death was confirmed by decapitation. Each individual was photographed, measured, and weighed. Detailed information on individual fish is provided in Table S1. All procedures were approved by the animal ethics committee of the University of Namur (UN PM KE 23/020). Following euthanasia, brains were collected and stored at −80°C until DNA extraction.

### 2.2 Reduced‐Representation Bisulfite Sequencing of Brain Samples

#### 2.2.1 DNA Extraction

DNA was extracted using NucleoSpin Tissue XS kit (Marcherey‐Nagel, item number 740901.250), with slight modifications to the manufacturer's protocol. An additional wash step, identical to the first, was included, and a 5‐min incubation at 37°C was added following the binding buffer step. Elution was performed using 2 × 10 μL (instead of once 20 μL), with each followed by centrifugation at 11,000 × *g* for 1 min. DNA quality and quantity were assessed via both a NanoDrop spectrophotometer and a high‐intensity Qubit fluorimeter. Samples that did not meet the minimum amount of DNA required for the Diagenode RRBS kit (< 50 ng of DNA, see below) were excluded from further analyzes (two samples in total).

#### 2.2.2 Reduced‐Representation Bisulfite Sequencing

RRBS libraries were prepared via the Premium RRBS Kit V2 from Diagenode (catalog number C02030037) following the manufacturer's protocol. This kit includes all the reagents required for enzymatic digestion, library preparation, bisulfite conversion, and PCR amplification. The final DNA concentrations were determined via a high‐sensitivity Qubit fluorimeter. Libraries were sequenced on a NovaSeq 6000 V1.5 (300 cycles XP workflow) with paired‐end sequencing (20 million reads each way) via the GIGA platform (University of Liège, Belgium). The FASTQ files were quality checked with FastQC v0.11.8 (https://www.bioinformatics.babraham.ac.uk/projects/fastqc/).

### 2.3 Bioinformatics

#### 2.3.1 Methylation Analysis

RAW FASTQ files were trimmed using *Trim Galore!* (version 0.6.10) with the following parameters: ‐rrbs ‐quality 28 ‐illumina ‐stringency 2 ‐length 40. Genome indexing and bisulfite alignment were performed using *Bismark* (v. 0.24.1). The latest version of the reference genome of *K. marmoratus* was used (GCF_001649575.2_ASM164957v2) and prepared via *Bismark_genome_preparation*. Read alignment was conducted via *Bowtie2* through *Bismark* with the following parameters: ‐score_min L,0, 0.6. *SAMtools* (version 1.17) was then used to sort and index the resulting BAM files.

The files were then processed in R (version 4.2.2) via the *methylKit* (version 1.28.0) and *caret* (version 6.0–94) packages. Bam files were processed with *processBismarkAln* via assembly = ASM164957v2 and read.context = “CpG”. Coverage filtering was applied via *filterByCoverage*, which retained only sites with a minimum coverage of 15 and maximum coverage below 99.9th percentile to mitigate PCR bias.

#### 2.3.2 Predicting Age From CpG Methylation

**FIGURE 1:**
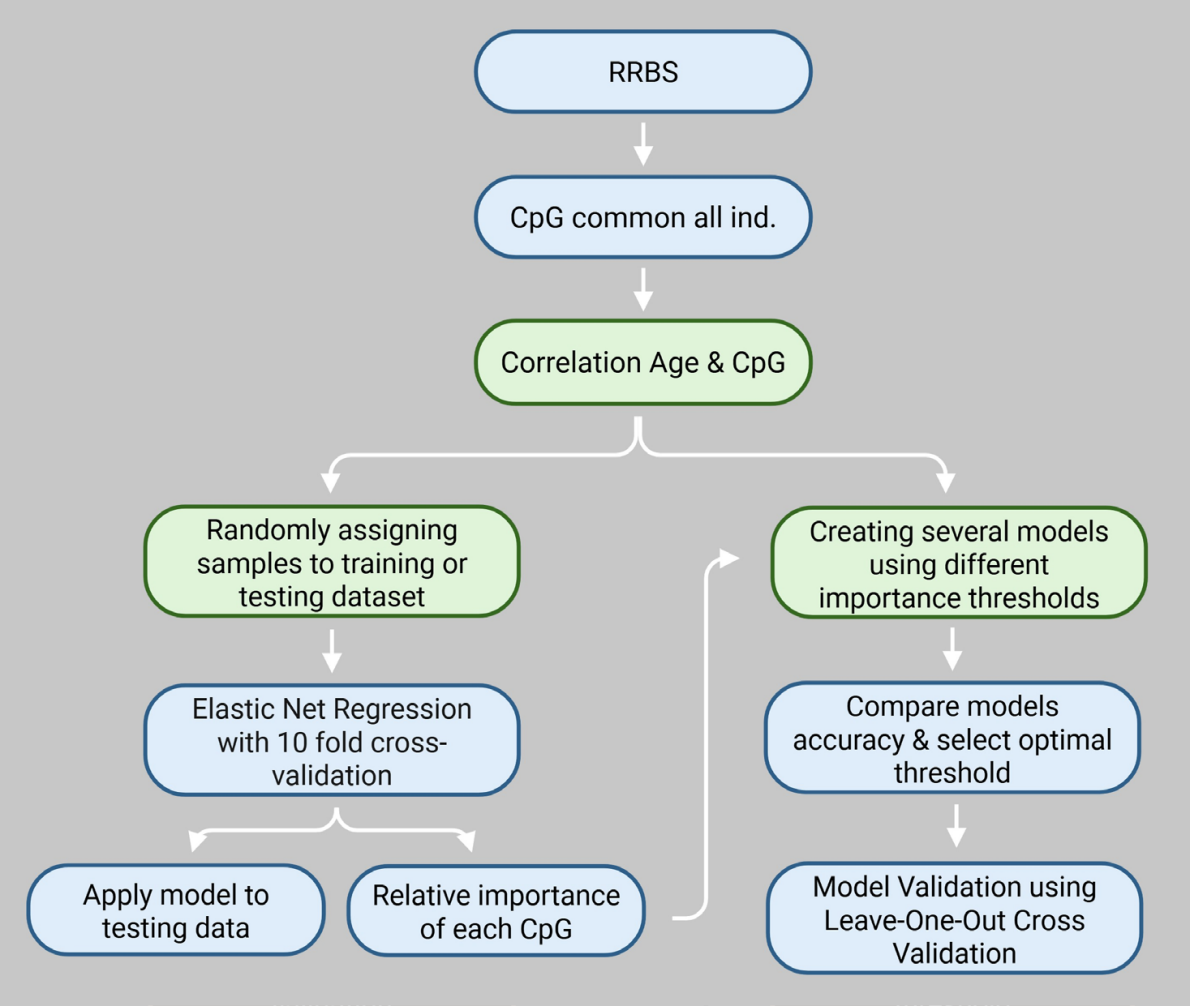
Diagram of the main stages in building and validating the model. The green frames are steps that have been studied and optimized. Their conclusions are discussed in the materials and methods section.

The model was built and validated following the main steps shown in Figure 1. The CpGs present in all the samples were first selected using *methylKit::unite*. The *percMethylation* function was used to create the percentage methylation matrix. The first selection of CpGs was carried out by correlating each CpG with the logarithm of the age of the individuals (*cor.test*, with Benjamini–Hochberg correction). Age was transformed to a natural log to fit a linear model. Preselecting the CpG stabilizes the sites selected in the final model when several seeds are compared. Preselection of informative features in high‐throughput datasets has been shown to improve model performance (Newediuk et al. 2025). By eliminating uninformative predictors at an early stage, feature selection reduces overfitting and enhances prediction accuracy on independent datasets (Theng et al. 2025).

The samples were randomly assigned to either a training (80%) or a testing dataset (20%) via the *createDataPartition* function in the caret R package. This division is necessary to prevent false improvements in model predictions. We tested the most common division, 70–30, 75–25, and 80–20, and compared the model accuracy with three different seeds. The 80–20 partition always provided the best results (Table S2).

The *training* function from the *caret* package was used with 10‐fold cross‐validation to perform elastic net regression on the training dataset. The use of elastic net regression is optimized for high throughput data as the hyperparameters α and λ shrink uninformative sites to zero and remove them for the models (Kuhn and Johnson 2013). *caret* tests the combination of three α (0.1, 0.5 and 1) and λ (depending on the data, always between 0 and 1) values and selects the combination best suited to the data, i.e., the one with the highest *R* ^2^ (strength of linear relationship between chronological and epigenetic age) and lowest Median Absolute Error (MAE, precision of the clock's predictions) on the testing data.

To focus on biologically informative markers of brain aging, we further reduced the number of CpG sites by selecting an optimal trade‐off between model complexity and predictive performance. The elastic net model assigns each CpG a relative importance score (0–100), which we used to iteratively filter predictors. We first conducted a coarse screening using an importance threshold ranging from 5 to 75 in steps of 5, followed by a finer screening between 25 and 35 in steps of 1. The final model was selected based on achieving high predictive accuracy (*R* ^2^ > 0.95) with the smallest number of CpGs when evaluated on the testing dataset. This stepwise approach revealed a non‐linear relationship between the number of predictors and model performance, indicating that subsets of CpGs contribute unequally and are not strictly complementary.

**FIGURE 2:**
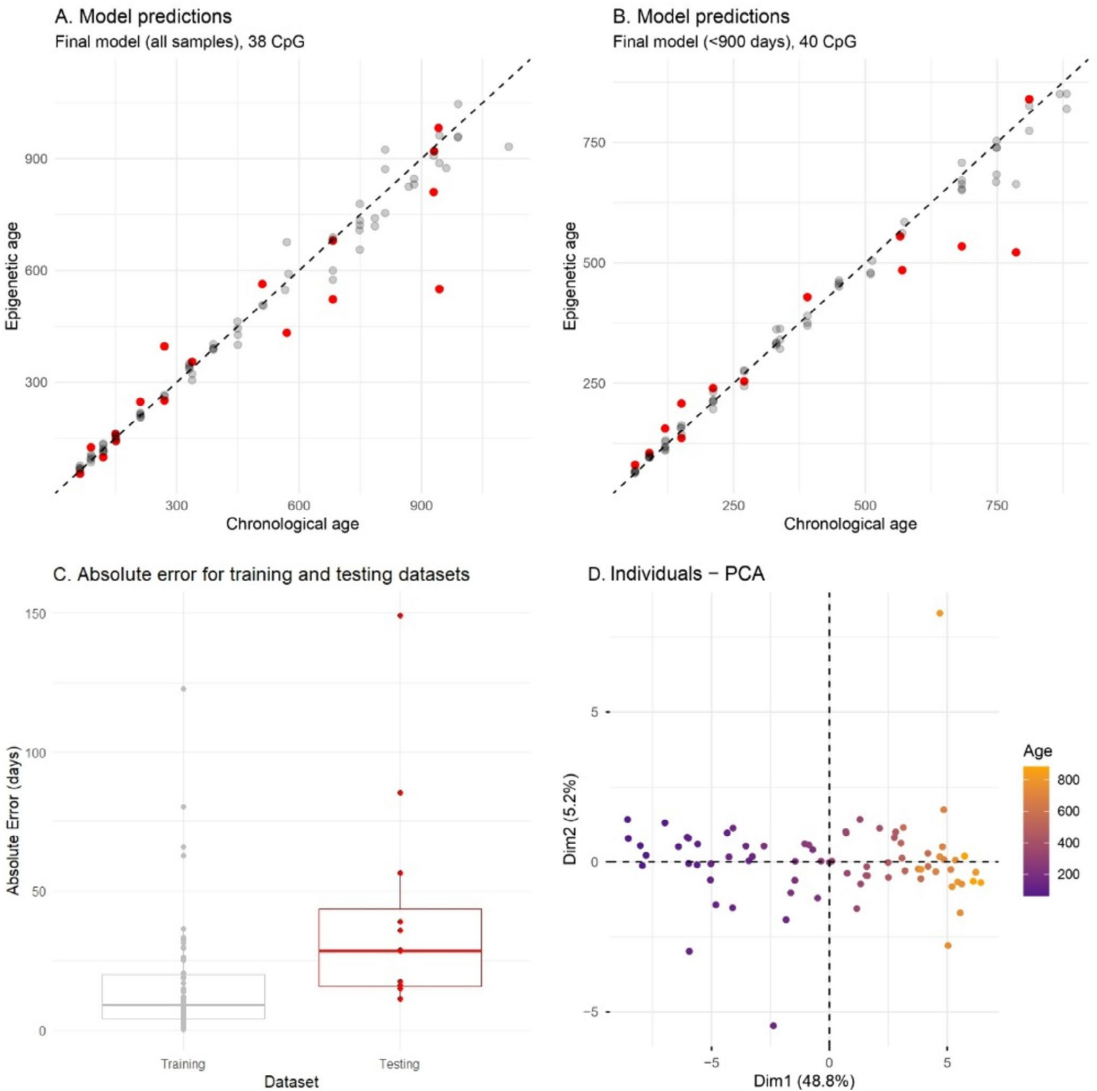
Mangrove rivulus age estimation. Chronological age is plotted against predicted age, for both training (in gray) and testing (in red) datasets, for both all individuals (A) and individuals younger than 900 days only (B). Absolute Error for both training and testing datasets, for the regression excluding the older individuals (C). PCA of individuals using the 40 CpG sites, showing a clear separation based on age in the first principal component (D).

The initial model was trained using all 89 individuals, yielding an *R* ^2^ of 0.937 and a median absolute error (MAE) of 35.6 days (Figure 2A). Because prediction accuracy decreased in older individuals, we repeated the entire modeling procedure using only the 76 individuals younger than 900 days. This age‐restricted model showed improved performance (*R* ^2^ = 0.96; MAE = 28.7 days; Figure 2B) and was therefore retained as the final epigenetic clock. Unless stated otherwise, all subsequent results and interpretations are based on this reduced model.

The model was ultimately evaluated using Leave‐One‐Out Cross Validation (LOOCV), where each fold consists of a single observation. In this approach, the model is trained on all data points except one, which is then used for testing. This process is repeated systematically so that every observation serves once as the test set. LOOCV provides an estimate of the true test error, as training is always performed on the full dataset minus one sample (James et al. 2013). The results revealed no difference between the model's performance and its validation.

PCA was then performed on methylation data using the *FactoMineR* package (version 2.10) to visualize the individuals with respect to their age.

### 2.4 Annotation

A file containing the chromosome, start and stop positions, and strand information for each selected CpG site was imported into *SeqMonk* (version 1.481) and analyzed using the genome assembly ASM164957v2 (Kelley et al. 2016). Probes were generated by assigning CpG sites to genes located within a maximum distance of 20,000 base pairs. The resulting annotation file included, in addition to the imported data, the associated gene name (*Features*), the Feature Orientation (upstream, overlapping, downstream, or null), and the distance between the CpG site and the gene when not overlapping. The roles of the genes were described via Z‐fins to maintain consistency in the annotations.

## 3 Results

### 3.1 Age‐Associated CpG Sites Identified by RRBS

Information about the fish used (Fish ID, Age at death and Length) is in Table S1. The bisulfite conversion was 98.87%. RRBS samples were aligned to the *K. marmoratus* reference genome, with an average alignment rate of 56.8%. The percentage of global methylation was 60.2%. Global methylation did not significantly correlate with age (*p* value = 0.5748, Figure S1).

A total of 467,182 CpG sites were common to all individuals, with a minimum coverage of 15 and maximum coverage set at the 99.9th percentile. Among these sites, 36,704 CpGs were significantly correlated with age (correlation test, corrected for multiple testing via the Benjamini‐Hochberg procedure). The selected regression, using 80/20 split, has an α value of 0.1 and a λ value of 0.05. This model achieved an *R* ^2^ of 0.938 and MAE of 35.6 days, using 38 CpG. However, as the correlation was less accurate in older individuals (Figure 2A), a second model was rerun using only individuals younger than 900 days (76 individuals). A total of 475,502 CpG sites were common to these individuals, with a minimum coverage of 15 and maximum coverage set at the 99.9th percentile. Among these sites, 29,126 were significantly correlated with age (correlation test, corrected for multiple testing via the Benjamini–Hochberg procedure). The selected regression has an α value of 0.1 and a λ value of 0.150. The final model included 40 CpG sites, achieved an *R* ^2^ of 0.960 and MAE of 28.7 days (Figure 2B).

Validation of the model by Leave‐One‐Out‐Cross‐Validation (LOOCV) yielded virtually identical results, indicating that both approaches capture true accuracy equally well. LOOCV of both models is in Figure S2. A MAE of 28.7 days was found in the testing dataset, and no statistical difference was observed between the absolute error rate between the training and testing data sets (*p*‐value = 0.066, welch‐test, two‐tailed) (Figure 2C).

We subsequently performed a PCA of the individuals based on their methylation profile (Figure 2D), with chronological age represented as a color gradient. The first principal component explained 48.8% of the total variance and was strongly associated with age. The second component explained 5.2% of the total variance.

### 3.2 Identification and Characterization of Age‐Related CpG Sites

Age‐associated DNA methylation levels are often highly correlated across CpG sites. In the presence of multicollinearity, elastic net regression selects one predictor arbitrarily from a set of correlated CpGs, leading to variability in the specific sites retained across repeated model fits (Engebretsen and Bohlin 2019). Accordingly, annotation of individual CpG sites included in the final model should not be interpreted as uniquely informative or causal (Moqri et al. 2023), but rather to provide genomic context.

Information for each CpG site and its associated gene is summarized in Table 1. Among the 40 selected CpG sites, 31 showed an increase in the percentage of methylation with age, whereas 9 exhibited a decrease. Among these sites, 31 were located within gene bodies, 8 were positioned upstream (ranging from 232 to 5622 base pairs from the start codon), and 1 was located downstream (11,307 base pairs). Each gene was evaluated for methylation level changes (DNAm change), strength of association (*R* ^2^), genomic context, distance to the gene if not within it, and functional relevance (Table 1). The age‐associated methylation trajectories of individual CpG sites display substantial heterogeneity and are shown in Figure S3.

**TABLE 1:** Chromosome and position, distance to the gene, *R* ^2^ and DNA methylation change (from 60 DPH to 900 DPH), gene name and ID and biological significance using Z‐fin.

Chr, position	Distance	*R* ^2^, DNAm change	Gene name and ID	Biological significance (Z‐fin)
Chr1, 9323024	1291	0.566 19%→43%	gnb1l Guanine nucleotide‐binding protein subunit beta‐like protein 1 ZDB‐GENE‐050320‐77	Involved in cell cycle progression, signal transduction, apoptosis, and gene regulation.
Chr1, 10730302	0	0.546 50%→75%	msi1b musashi RNA binding protein 1b ZDB‐GENE‐050320‐86	Enable mRNA binding activity.
Chr2, 7418712	0	0.475 52%→79%	synrg synergin ZDB‐GENE‐030131‐9396	Part of clathrin coat of trans‐Golgi network vesicle.
Chr3, 14648133	0	0.683 26%→56%	lrba LPS‐responsive vesicle trafficking, beach and anchor containing ZDB‐GENE‐090311‐19	Enable protein kinase binding activity. Involved in protein localization.
Chr4, 11103886	0	0.267 14%→26%	hyal1 hyaluronidase 1 ZDB‐GENE‐060312‐42	Enable hyalurononglucosaminidase activity. Involved in hyaluronan catabolic process.
Chr5, 1055068	0	0.28 28%→42%	lmna lamin A ZDB‐GENE‐020424‐3	Structural constituent of cytoskeleton.
Chr5, 6018182	0	0.214 15%→32%	skiv2l Ski2‐like RNA Helicase ZDB‐TSCRIPT‐100915‐49	Enable RNA helicase activity. Involved in nuclear‐transcribed mRNA catabolic process, 3′‐5′ exonucleolytic nonsense‐mediated decay.
Chr5, 10638440	0	0.423 6%→20%	klhl38a kelch‐like family member 38a ZDB‐GENE‐060503‐737	Enable ubiquitin‐like ligase‐substrate adaptor activity. Involved in proteasome‐mediated ubiquitin‐dependent protein catabolic process. Part of Cul3‐RING ubiquitin ligase complex.
Chr6, 181223	0	0.378 56%→32%	TTN Titin ZDB‐GENE‐030616‐413	Structural constituent of muscle. Acts upstream of or within several processes, including heart contraction and striated muscle tissue development.
Chr6, 2171690	0	0.34 93%→74%	cops8 COP9 signalosome subunit 8 ZDB‐GENE‐040426‐982	Act upstream of or within COP9 signalosome assembly and protein deneddylation.
Chr6, 2171717	0	0.214 95%→86%		
Chr6, 19162718	232	0.513 54%→80%	Ahr aryl hydrocarbon receptor ZDB‐GENE‐990714‐16	Enables nuclear receptor activity and sequence‐specific DNA binding activity. Acts upstream of or within several processes, including fin development; ovarian follicle development; and positive regulation of transcription by RNA polymerase II.
Chr8, 12560302	0	0.425 10%→25%	IQSEC1 IQ motif and Sec7 domain ArfGEF 1 ZDB‐GENE‐120813‐2	Enable guanyl‐nucleotide exchange factor activity. Acts upstream of or within angiogenesis and lymphangiogenesis.
Chr8, 13059272	0	0.269 5%→15%	plxnd1 plexin D1 ZDB‐GENE‐040426‐1828	Enable semaphorin receptor activity. Acts upstream of or within with a negative effect on sprouting angiogenesis. Acts upstream of or within embryonic eye morphogenesis and vasculature development.
Chr10, 9160868	1174	0.413 13%→35%	fermt2 FERM domain containing kindlin 2 ZDB‐GENE‐050506‐132	Enable integrin binding activity. Acts upstream of or within cell migration; circulatory system development; and cytoskeleton organization.
Chr11, 12833818	0	0.437 46%→23%	slc25a22a solute carrier family 25‐member 22a ZDB‐GENE‐110408‐61	Enable L‐aspartate transmembrane transporter activity and L‐glutamate transmembrane transporter activity.
Chr11, 12950935	0	0.537 17%→35%	Saxo4/ppp1r32 stabilizer of axonemal microtubules 4 ZDB‐GENE‐120913‐1	Enable phosphatase binding activity.
Chr11, 14053781	0	0,456 63%→86%	otud7a OTU deubiquitinase 7A ZDB‐GENE‐100212‐1	Enable DNA binding activity; cysteine‐type deubiquitinase activity; and zinc ion binding activity.
Chr14, 2182519	0	0.589 12%→26%	tfap2e transcription factor AP‐2 epsilon ZDB‐GENE‐040426‐1455	Enable DNA‐binding transcription activator activity, RNA polymerase II‐specific and RNA polymerase II transcription regulatory region sequence‐specific DNA binding activity. Acts upstream of or within melanocyte differentiation.
Chr14, 2183494	0	0.608 30%→60%		
Chr14, 7714032	5622	0.511 59%→81%	myo18b myosin XVIIIB ZDB‐GENE‐111111‐8	Acts upstream of or within sarcomerogenesis.
Chr15, 5068118	0	0.539 40%→66%	cdh15 cadherin 15 ZDB‐GENE‐030131‐780	Enable beta‐catenin binding activity and cadherin binding activity. Involved in several processes, including calcium‐dependent cell–cell adhesion via plasma membrane cell adhesion molecules; cell–cell adhesion mediated by cadherin; and cell–cell junction organization
Chr16, 10652845	0	0.618 50%→10%	dbpb D site albumin promoter binding protein b ZDB‐GENE‐100922‐6	Enable DNA‐binding transcription factor activity, RNA polymerase II‐specific and RNA polymerase II cis‐regulatory region sequence‐specific DNA binding activity. Involved in regulation of transcription by RNA polymerase II. Act upstream of or within regulation of DNA‐templated transcription.
Chr16, 11363278	0	0.358 40%→20%	itga10 integrin, α 10 ZDB‐GENE‐100922‐54	Enable integrin binding activity. Involved in several processes, including cell adhesion mediated by integrin; cell‐matrix adhesion; and integrin‐mediated signaling pathway. Act upstream of or within cell adhesion.
Chr16, 14332044	673	0.627 10%→33%	twist1b twist family bHLH transcription factor 1b ZDB‐GENE‐050417‐357	Enable cis‐regulatory region sequence‐specific DNA binding activity. Acts upstream of or within several processes, including circulatory system development; pharyngeal system development; and regulation of bone mineralization.
Chr16, 29541652	0	0.562 60%→32%	cep162 centrosomal protein 162 ZDB‐GENE‐100922‐266	Involved in cilium assembly. Acts upstream of or within heart looping.
Chr17, 13385366	2958	0.615 27%→54%	tut1 terminal uridylyl transferase 1, U6 snRNA‐specific ZDB‐GENE‐050706‐68	Enable enzyme‐substrate adaptor activity; mRNA 3′‐UTR binding activity; and nucleotidyltransferase activity. Involved in cotranscriptional mRNA 3′‐end processing, cleavage and polyadenylation pathway and snRNA processing. Act upstream of or within mRNA processing.
Chr18, 15967531	998	0.652 63%→91%	ascl4 Achaete‐Scute Family BHLH Transcription Factor 4 MGI:1914591^a^	Enable DNA‐binding transcription factor activity, RNA polymerase II‐specific and RNA polymerase II transcription regulatory region sequence‐specific DNA binding activity. Involved in regulation of transcription by RNA polymerase II.^a^
Chr19, 265466	0	0.777 38%→78%	nkx2.4b NK2 homeobox 4b ZDB‐GENE‐000830‐1	Enable DNA‐binding transcription factor activity, RNA polymerase II‐specific and RNA polymerase II cis‐regulatory region sequence‐specific DNA binding activity. Acts upstream of or within hypothalamus development and thyroid gland development.
Chr19, 265551	0	0.677 30%→70%		
Chr19, 265783	0	0.489 52%→80%		
Chr19, 15280333	2755	0.558 50%→76%	peli2 pellino E3 ubiquitin protein ligase family member 2 ZDB‐GENE‐040718‐360	Enable ubiquitin protein ligase activity. Predicted to be involved in protein polyubiquitination. Act upstream of or within regulation of Toll signaling pathway.
Chr20, 3209495	0	0.426 32%→60%	jcad junctional cadherin 5 associated a ZDB‐GENE‐111221‐2	Involved in positive regulation of blood vessel endothelial cell proliferation involved in sprouting angiogenesis.
Chr20, 10412132	−11,307	0.584 35%→65%	cdh2 cadherin 2, type 1, N‐cadherin ZDB‐GENE‐990415‐171	Enable beta‐catenin binding activity; cadherin binding activity; and calcium ion binding activity. Involved in cell–cell adhesion and neural tube formation.
Chr20, 11307320	0	0.393 60%→80%	tmem275 transmembrane protein 275 ZDB‐GENE‐131121‐130	Located in membrane.
Chr21, 15660274	0	0.401 34%→6%	map3k22 mitogen‐activated protein kinase kinase kinase 22 ZDB‐GENE‐070928‐11	Enable protein serine/threonine kinase activity. Involved in intracellular signal transduction. Act upstream of or within protein phosphorylation.
Chr22, 26875602	0	0.521 30%→57%	Srfb serum response factor b ZDB‐GENE‐040426‐1294	Enable DNA‐binding transcription factor activity, RNA polymerase II specific; cis‐regulatory region sequence‐specific DNA binding activity; and protein dimerization activity. Act upstream of or within positive regulation of transcription by RNA polymerase II.
Chr24, 12606753	0	0.467 3%→22%	haus8 HAUS augmin like complex subunit 8 ZDB‐GENE‐030131‐6830	Enable microtubule binding activity. Involved in centrosome cycle and spindle assembly.
Chr24, 12606769	0	0.547 8%→40%		
Chr24, 24522970	0	0.275 45%→25%	ltbp latent transforming growth factor beta binding protein ZDB‐GENE‐091202‐8	Involved in transforming growth factor beta receptor signaling pathway. Acts upstream of or within several processes, including heart morphogenesis; regulation of smooth muscle tissue development; and smooth muscle cell proliferation.

^a^ Not found on Z‐fin. From NCBI, in *Mus musculus* .

The CpG sites with the highest *R* ^2^ values are within the genes nkx2.4b (position 265466, *R* ^2^ of 0.777), lrba (*R* ^2^ of 0.683), nkx2.4b (position 265551, *R* ^2^ of 0.677), ascl4 (*R* ^2^ of 0.652) and dbpb (0.618). Their methylation levels changed by at least 30%, with an increase for the first four and a decrease for dbpb (50%–10%). The nkx2.4b gene appears twice in the 5 most correlated CpG sites, and a third CpG site present in this gene was also selected within our 40 CpG sites, with an *R* ^2^ of 0.489. Two CpG sites were also selected within the same gene for cops8 (*R* ^2^ values of 0.340 and 0.214, both decreasing methylation), haus8 (*R* ^2^ values of 0.547 and 0.467, both increasing methylation), and tfap2e (*R* ^2^ values of 0.608 and 0.589, both increasing methylation).

Among the genes found, several essential functions were identified. Notably, cell cycle progression, signal progression, and apoptosis (gnb1l), mRNA binding activity (msi1b), mRNA 3′‐end processing, cleavage and polyadenylation (tut1), protein kinase activity (lrba), cytoskeleton (lmna), structural component of muscle (titin), microtubule binding activity (haus8), DNA binding transcription activity (tfap2e, dbpb, nkx2.4b, and srfb), serine/threonine kinase activity (map3k22), and transformation of the growth factor beta receptor signaling pathway (ltbp) were investigated. The complete list of gene roles associated with the selected CpG sites can be found in Table 1.

## 4 Discussion

In this study, we developed the first brain‐based epigenetic clock for a teleost fish. Using the mangrove rivulus *Kryptolebias marmoratus*, we investigated non‐pathological brain aging in a system characterized by extremely low genetic variation (Taylor 2000). *K. marmoratus* exhibits an androdioecious mating system composed of self‐fertilizing hermaphrodites and males, generating naturally occurring near‐isogenic lineages. Self‐fertilization dramatically reduces genetic variation among individuals, providing a powerful framework to disentangle age‐associated epigenetic changes from genetic background effects (Kelley et al. 2016). These features make *K. marmoratus* a uniquely suited model for studying epigenetic aging with minimal genetic confounding.

Beyond constructing an epigenetic age predictor in a self‐fertilizing vertebrate, we aimed to characterize baseline epigenetic aging dynamics in the brain. We identified 40 CpG sites whose combined methylation profiles predict chronological age with high accuracy (*R* ^2^ > 0.96; mean absolute error = 28.7 days). Among genes associated with the selected CpGs, several have attracted our attention in the study of aging. It is important to underline that the selected CpGs should not be interpreted as uniquely determining aging‐related processes or as having direct functional roles (Moqri et al. 2023). Gene annotations associated with these CpG sites are therefore presented to provide biological context and to facilitate comparison with aging‐related pathways described in other systems, rather than to imply a causal relationship between methylation at individual loci and gene function. Nevertheless, these associations provide a foundation for future experimental studies to test causal relationships between specific methylation changes and aging phenotypes. Exploring the broader landscape of age‐correlated CpGs through enrichment analysis could provide valuable complementary insights into the biological pathways underlying aging in this species.

The CpG at position 1055068 on chromosome 5 is within the gene *lamin‐A* (*lmna*) and shows hypermethylation with age (from 28% of methylation at 60 days to 42% at 900 days). *lmna* encodes the prelamin‐A/C protein, which undergoes four post‐translational steps to become mature lamin A, the main constituent of the nuclear lamina (Lin and Worman 1993). A defect in the post‐translational maturation disrupts nuclear function and affects aging‐related processes, such as mTOR signaling, epigenetic modifications, the stress response, inflammation, microRNA activation, and mechanosignaling, causing premature aging syndromes such as Hutchinson–Gilford progeria syndrome (Cenni et al. 2020). Moreover, lamin A, which is typically not expressed in neurons, appears to transform from senile to Alzheimer's disease (AD) neurons and contributes to halting the consequences of cell cycle re‐entry and nuclear Tau exit, allowing the survival of the neuron. This irreversible nuclear transformation leads to neurofibrillary tangle formation and, ultimately, neurodegeneration (Gil et al. 2021).

A second CpG site of interest is located at position 19162718 on chromosome 6, 232 base pairs upstream from the gene *Aryl Hydrocarbon Receptor* (AhR). It shows hypermethylation with age, ranging from 54% to 80%. AhR is a ligand‐dependent transcription factor that is classically associated with the regulation of xenobiotic metabolism in response to environmental toxicants (Hankinson 1995). Several AhR ligands, such as resveratrol and quercetin, have been shown to extend the lifespan of model organisms (Bhullar and Hubbard 2015; Kampkötter et al. 2008; Pietsch et al. 2009). However, conflicting results have been obtained, depending on the ligand, species, and experimental setup used (Eckers et al. 2016; Serna et al. 2024). It has also been suggested that AhR reveals evidence of antagonistic pleiotropy in the regulation of the aging process, i.e., the gene is beneficial during development but shows deleterious properties during aging (Salminen 2022).

Two selected CpG sites are within *cops8* (COP9 signalosome 8) and are both hypomethylated with age (position 2171690, ranging from 93% methylation to 74%, and position 2171717, ranging from 95% to 86%). The COP9 signalosome participates in several cellular and developmental processes in various eukaryotic organisms and is associated with de‐ubiquitination and protein kinase activities (Wei and Deng 2003). Subunit 8 is a central regulator of the proteasomal proteolytic pathway and selective autophagy (Su et al. 2011). The autophagic–lysosomal pathway is critical for the removal of oxidized proteins in the heart (Su et al. 2013). Like epigenetic alterations, impaired autophagy is one of the primary hallmarks of aging (López‐Otín et al. 2023).

Among the other selected CpGs, several show a link with AD. A Genome Wide Association study revealed that FERM domain containing kindlin 2 (*fermt2*) (CpG 1174 base pair upstream, which is hypermethylated with age) is a locus susceptible to AD. These results suggested that *fermt2* modulates AD risk by regulating APP metabolism and Aβ peptide production (Chapuis et al. 2017). Another GWAS revealed that an increased risk of developing AD has been associated with 27 genes, including *fermt2*. LPS‐responsive vesicle trafficking, beach and anchor containing (*Lrba*), solute carrier family 25‐member 22a (*slc25A22*), and integrin α 10 (*itga10*) are differentially expressed in AD patients compared with healthy cognitive adults and have been found in our epigenetic clock (Kim et al. 2023; Tian et al. 2024; Zou et al. 2023).

Our study identified molecular markers of baseline brain aging in the mangrove rivulus that are also implicated in age‐related neurological diseases in humans. This overlap highlights the potential of experimentally perturbing these markers to assess their causal roles in pathological aging. Such perturbations could be achieved through controlled exposure to neurotoxic compounds or via targeted genetic and epigenetic manipulations, including CRISPR–Cas9 and CRISPR–dCas9–based approaches (Cai et al. 2023; Caobi et al. 2020; Chen et al. 2018; Ruetz et al. 2024). Although we have focused on CpGs associated with genes that have been linked in literature to aging and/or neurodegeneration, it is important to study the mechanism of other CpGs in the future. A particular focus should be placed on the *nkx2.4b*, *haus8*, and *tfap2e* genes, for which several CpGs have been selected. These genes may have an underestimated role in the aging and death of individuals, but no literature has mentioned them. Exploring these candidates not only broadens our understanding of potential contributors to aging but also provides an opportunity to investigate how their alteration may drive or protect against disease processes.

The selected CpG sites and the sequencing data generated add to the data already available for other teleost species, e.g., European sea bass (Anastasiadi and Piferrer 2020), scorpion fish (Weber et al. 2022), cow nose rays (Weber et al. 2024) and haddock (Strand et al. 2025), all of which show very high accuracy. The increasing number of studies on the teleost epigenetic clock has increased interest in the construction of a pan‐teleost epigenetic clock, which would be particularly valuable in the fields of aquaculture and conservation (Piferrer and Anastasiadi 2023). A first pan‐mammalian epigenetic clock has been built, including 185 species and 59 tissue types, and show very high accuracy (*r* > 0.96) (Lu et al. 2023). This pan‐epigenetic clock proposes solutions to solve the inconstancy in sequencing methods, different lifespans, etc., underlying the feasibility of such a teleost epigenetic clock. Other prospects include applying this epigenetic clock to other mangrove rivulus lineages, with various genetic diversity, as well as natural populations. This method would provide valuable ecological information by enabling the estimation of age structures in natural populations, thereby deepening our understanding of demographic processes and life history traits (Piferrer and Anastasiadi 2023).

Many epigenetic clocks have been described in the literature; however, there remains little consensus regarding key regression parameters used in their construction. In this study, we compared different modeling choices to assess their influence on predictive performance within our dataset. Notably, the 80–20 split between training and testing data consistently yielded superior performance on the testing dataset (Table S2). To avoid the risk of overfitting (artificially inflating model performance), we encouraged implementing a 10‐fold cross‐validation procedure, which provides a more robust estimate of model generalizability by evaluating performance across multiple training–testing splits within the training data. Furthermore, we encourage a first selection of the predictor based on their own correlation with age, as it reduces model complexity and improves stability (Engebretsen and Bohlin 2019). Prioritizing CpG with stronger individual age association improves interpretability and better reflects selected biological processes involving specific epigenetic features (Bertucci and Parrott 2020). Regarding the final CpG selection, we noticed a non‐linear relationship between the number of CpG sites included and predictive accuracy; therefore, we recommend a careful evaluation of the inclusion threshold to achieve a parsimonious model with stable performance.

As has been seen in other work (e.g., El Khoury et al. 2019), our epigenetic clock tends to underestimate chronological age in older individuals. One hypothesis for this bias is early saturation of methylation levels at selected CpG sites, such that the regression model extrapolates to biologically implausible methylation values. Another contributing factor may be the presence of 5‐hydroxymethylcytosine (5hmC), which cannot be distinguished from 5‐methylcytosine (5mC) using conventional bisulfite sequencing and may distort age predictions in tissues with high 5hmC abundance, such as the brain (El Khoury et al. 2019). Although the level of 5hmC in mangrove rivulus brain tissue has not yet been characterized, studies in other teleost fish indicate that hydroxymethylation plays an important regulatory role. In *Oreochromis niloticus* , differential hydroxymethylation has been reported in genes associated with growth, particularly within gene bodies and promoters (Konstantinidis et al. 2023). Age‐associated epigenetic drift may further contribute to reduced predictive accuracy in older individuals (Hannum et al. 2013; Heyn et al. 2012).

In conclusion, our study not only revealed age‐related changes in CpG methylation patterns but also represents the first development of an epigenetic clock in a self‐fertilizing vertebrate, the mangrove rivulus. This unique model allows the study of DNAm changes without the confounding effects of genetic variation, providing insights into the specific roles of DNAm in aging. Using brain tissue enabled us to investigate baseline aging processes in this organ and to gain functional insights into brain aging. The genes identified here warrant further investigation to improve our understanding of brain aging and its links to neurodegenerative diseases.

## Author Contributions

**Justine Bélik:** conceptualization (equal), data curation (lead), formal analysis (lead), funding acquisition (supporting), methodology (lead), visualization (lead), writing – original draft (lead), writing – review and editing (equal). **Frédéric Silvestre:** conceptualization (equal), funding acquisition (lead), methodology (supporting), supervision (lead), validation (lead), writing – review and editing (equal).

## Funding

This work was supported by Fonds De La Recherche Scientifique – FNRS (Grant J.0189.24). Computational resources have been provided by the Consortium des Équipements de Calcul Intensif (CÉCI), funded by the Fonds de la Recherche Scientifique de Belgique (F.R.S.‐FNRS) under Grant No. 2.5020.11 and by the Walloon Region.

## Ethics Statement

All research reported in this manuscript was conducted in accordance with institutional and national ethical standards for animal care and use. Experimental procedures involving *Kryptolebias marmoratus* were approved by the Animal Experimentation Ethics Committee (UN PM KE 23/020).

## Consent

The authors have nothing to report.

## Conflicts of Interest

The authors declare no conflicts of interest.

## Supporting information


**Figure S1:** Global methylation level with age.

**Figure S2:** Leave‐one‐out cross validation for both final models (all samples included on the left, only samples < 900 days on the right).

**Figure S3:** DNA methylation level of 40 selected CpG sites across chronological age.

**Table S1:** Identity (ID), age at death (days), and length (mm) of the fish used.
**Table S2:** Comparison of the 70–30, 75–25, and 80–20 divisions on 3 different seed, with the number of CpG and respective *R* ^2^. When no cut‐off offers exactly 40 CpGs, the number of CpGs was chosen to be as close to 40 as possible.

## Data Availability

The datasets generated and/or analyzed during the current study are available in the NCBI repository, under the ID *BioProject ID PRJNA1331489*. The code is available on the following GitHub page: https://github.com/jubelik/Epigenetic‐clock and Zenodo repository https://doi.org/10.5281/zenodo.20491333.

## References

[ece373881-bib-0001] Anastasiadi, D. , and F. Piferrer . 2020. “A Clockwork Fish: Age Prediction Using DNA Methylation‐Based Biomarkers in the European Seabass.” Molecular Ecology Resources 20, no. 2: 387–397. 10.1111/1755-0998.13111. 31674713

[ece373881-bib-0002] Andraszek, K. , M. Gryzińska , E. Wójcik , S. Knaga , and E. Smalec . 2014. “Age‐Dependent Change in the Morphology of Nucleoli and Methylation of Genes of the Nucleolar Organizer Region in Japanese Quail ( *Coturnix japonica* ) Model (Temminck and Schlegel, 1849) (Galliformes: Aves).” Folia Biologica 62, no. 4: 293–300. 10.3409/fb62_4.293. 25916156

[ece373881-bib-0086] Bertucci, E. M. , and B. B. Parrott . 2020. “Is CpG Density the Link between Epigenetic Aging and Lifespan?” Trends in Genetics 36, no. 10: 725–727. 10.1016/j.tig.2020.06.003. 32624337

[ece373881-bib-0003] Bertucci‐Richter, E. M. , and B. B. Parrott . 2023. “The Rate of Epigenetic Drift Scales With Maximum Lifespan Across Mammals.” Nature Communications 14, no. 1: 7731. 10.1038/s41467-023-43417-6. PMC10676422 38007590

[ece373881-bib-0004] Bhullar, K. S. , and B. P. Hubbard . 2015. “Lifespan and Healthspan Extension by Resveratrol.” Biochimica et Biophysica Acta, Molecular Basis of Disease 1852, no. 6: 1209–1218. 10.1016/j.bbadis.2015.01.012. 25640851

[ece373881-bib-0005] Bock, S. L. , K. Lyons , L. Yang , et al. 2026. “Genome‐Wide DNA Methylation Patterns Predict Age in the Zebra Shark (*Stegostoma tigrinum*) and Provide Insight Into the Evolution of Vertebrate Aging.” Molecular Ecology 35, no. 7: e70326. 10.1111/mec.70326. 41930734 PMC13047888

[ece373881-bib-0006] Brink, K. , C. L. Thomas , A. Jones , T. W. Chan , and E. B. Mallon . 2024. “Exploring the Ageing Methylome in the Model Insect, *Nasonia vitripennis* .” BMC Genomics 25, no. 1: 305. 10.1186/s12864-024-10211-7. 38519892 PMC10958858

[ece373881-bib-0007] Cai, R. , R. Lv , X. Shi , G. Yang , and J. Jin . 2023. “CRISPR/dCas9 Tools: Epigenetic Mechanism and Application in Gene Transcriptional Regulation.” International Journal of Molecular Sciences 24, no. 19: 14865. 10.3390/ijms241914865. 37834313 PMC10573330

[ece373881-bib-0008] Caobi, A. , R. K. Dutta , L. D. Garbinski , et al. 2020. “The Impact of CRISPR‐Cas9 on Age‐Related Disorders: From Pathology to Therapy.” Aging and Disease 11, no. 4: 895–915. 10.14336/AD.2019.0927. 32765953 PMC7390517

[ece373881-bib-0009] Caulton, A. , K. G. Dodds , K. M. McRae , C. Couldrey , S. Horvath , and S. M. Clarke . 2022. “Development of Epigenetic Clocks for Key Ruminant Species.” Genes 13, no. 1: 96. 10.3390/genes13010096. PMC8775075 35052436

[ece373881-bib-0010] Cenni, V. , C. Capanni , E. Mattioli , et al. 2020. “Lamin A Involvement in Ageing Processes.” Ageing Research Reviews 62: 101073. 10.1016/j.arr.2020.101073. 32446955

[ece373881-bib-0011] Chapelle, V. 2023. “Adaptation and Evolution With Low Genetic Diversity: A Combined Field and Laboratory Study on DNA Methylation Variation in the Mangrove Rivulus *Kryptolebias marmoratus*.”

[ece373881-bib-0012] Chapuis, J. , A. Flaig , B. Grenier‐Boley , et al. 2017. “Genome‐Wide, High‐Content siRNA Screening Identifies the Alzheimer's Genetic Risk Factor FERMT2 as a Major Modulator of APP Metabolism.” Acta Neuropathologica 133, no. 6: 955–966. 10.1007/s00401-016-1652-z. 27933404 PMC5427165

[ece373881-bib-0013] Chen, C.‐D. , E. Zeldich , Y. Li , A. Yuste , and C. R. Abraham . 2018. “Activation of the Anti‐Aging and Cognition‐Enhancing Gene Klotho by CRISPR‐dCas9 Transcriptional Effector Complex.” Journal of Molecular Neuroscience 64, no. 2: 175–184. 10.1007/s12031-017-1011-0. 29352444 PMC5826803

[ece373881-bib-0014] Coninx, E. , Y. C. Chew , X. Yang , et al. 2020. “Hippocampal and Cortical Tissue‐Specific Epigenetic Clocks Indicate an Increased Epigenetic Age in a Mouse Model for Alzheimer's Disease.” Aging 12, no. 20: 20817–20834. 10.18632/aging.104056. 33082299 PMC7655172

[ece373881-bib-0015] Costa, W. J. E. M. , S. M. Q. Lima , and R. Bartolette . 2010. “Androdioecy in Kryptolebias Killifish and the Evolution of Self‐Fertilizing Hermaphroditism.” Biological Journal of the Linnean Society 99, no. 2: 344–349. 10.1111/j.1095-8312.2009.01359.x.

[ece373881-bib-0016] Davletgildeeva, A. T. , and N. A. Kuznetsov . 2024. “The Role of DNMT Methyltransferases and TET Dioxygenases in the Maintenance of the DNA Methylation Level.” Biomolecules 14, no. 9: 1117. 10.3390/biom14091117. 39334883 PMC11430729

[ece373881-bib-0017] De Paoli‐Iseppi, R. , B. E. Deagle , C. R. McMahon , M. A. Hindell , J. L. Dickinson , and S. N. Jarman . 2017. “Measuring Animal Age With DNA Methylation: From Humans to Wild Animals.” Frontiers in Genetics 8: 106. 10.3389/fgene.2017.00106. 28878806 PMC5572392

[ece373881-bib-0018] Eckers, A. , S. Jakob , C. Heiss , et al. 2016. “The Aryl Hydrocarbon Receptor Promotes Aging Phenotypes Across Species.” Scientific Reports 6, no. 1: 19618. 10.1038/srep19618. 26790370 PMC4726214

[ece373881-bib-0019] El Khoury, L. Y. , T. Gorrie‐Stone , M. Smart , et al. 2019. “Systematic Underestimation of the Epigenetic Clock and Age Acceleration in Older Subjects.” Genome Biology 20, no. 1: 283. 10.1186/s13059-019-1810-4. 31847916 PMC6915902

[ece373881-bib-0020] Engebretsen, S. , and J. Bohlin . 2019. “Statistical Predictions With Glmnet.” Clinical Epigenetics 11, no. 1: 123. 10.1186/s13148-019-0730-1. 31443682 PMC6708235

[ece373881-bib-0021] Fasolino, M. , S. Liu , Y. Wang , and Z. Zhou . 2017. “Distinct Cellular and Molecular Environments Support Aging‐Related DNA Methylation Changes in the Substantia Nigra.” Epigenomics 9, no. 1: 21–31. 10.2217/epi-2016-0084. 27981856 PMC5514978

[ece373881-bib-0022] Fuke, C. , M. Shimabukuro , A. Petronis , et al. 2004. “Age Related Changes in 5‐Methylcytosine Content in Human Peripheral Leukocytes and Placentas: An HPLC‐Based Study.” Annals of Human Genetics 68, no. 3: 196–204. 10.1046/j.1529-8817.2004.00081.x. 15180700

[ece373881-bib-0023] Gil, L. , S. A. Niño , G. Capdeville , and M. E. Jiménez‐Capdeville . 2021. “Aging and Alzheimer's Disease Connection: Nuclear Tau and Lamin A.” Neuroscience Letters 749: 135741. 10.1016/j.neulet.2021.135741. 33610669

[ece373881-bib-0024] Grodstein, F. , B. Lemos , L. Yu , et al. 2021. “The Association of Epigenetic Clocks in Brain Tissue With Brain Pathologies and Common Aging Phenotypes.” Neurobiology of Disease 157: 105428. 10.1016/j.nbd.2021.105428. 34153464 PMC8373772

[ece373881-bib-0025] Gryzinska, M. , E. Blaszczak , A. Strachecka , and G. Jezewska‐Witkowska . 2013. “Analysis of Age‐Related Global DNA Methylation in Chicken.” Biochemical Genetics 51, no. 7: 554–563. 10.1007/s10528-013-9586-9. 23553491 PMC3712131

[ece373881-bib-0026] Guynes, K. , L. A. Sarre , A. M. Carrillo‐Baltodano , et al. 2024. “Annelid Methylomes Reveal Ancestral Developmental and Aging‐Associated Epigenetic Erosion Across Bilateria.” Genome Biology 25, no. 1: 204. 10.1186/s13059-024-03346-z. 39090757 PMC11292947

[ece373881-bib-0027] Hankinson, O. 1995. “The Aryl Hydrocarbon Receptor Complex.” Annual Review of Pharmacology and Toxicology 35: 307–340. 10.1146/annurev.pa.35.040195.001515. 7598497

[ece373881-bib-0028] Hannum, G. , J. Guinney , L. Zhao , et al. 2013. “Genome‐Wide Methylation Profiles Reveal Quantitative Views of Human Aging Rates.” Molecular Cell 49, no. 2: 359–367. 10.1016/j.molcel.2012.10.016. 23177740 PMC3780611

[ece373881-bib-0029] Heydenrych, M. J. , B. J. Saunders , M. Bunce , and S. N. Jarman . 2021. “Epigenetic Measurement of Key Vertebrate Population Biology Parameters.” Frontiers in Ecology and Evolution 9: 3. 10.3389/fevo.2021.617376.

[ece373881-bib-0030] Heyn, H. , N. Li , H. J. Ferreira , et al. 2012. “Distinct DNA Methylomes of Newborns and Centenarians.” Proceedings of the National Academy of Sciences of the United States of America 109, no. 26: 10522–10527. 10.1073/pnas.1120658109. 22689993 PMC3387108

[ece373881-bib-0031] Horvath, S. 2013. “DNA Methylation Age of Human Tissues and Cell Types.” Genome Biology 14, no. 10: 3156. 10.1186/gb-2013-14-10-r115. PMC4015143 24138928

[ece373881-bib-0032] Horvath, S. , J. A. Zoller , A. Haghani , et al. 2021. “Epigenetic Clock and Methylation Studies in the Rhesus Macaque.” GeroScience 43, no. 5: 2441–2453. 10.1007/s11357-021-00429-8. 34487267 PMC8599607

[ece373881-bib-0033] James, G. , D. Witten , T. Hastie , and R. Tibshirani . 2013. “Moving Beyond Linearity.” In An Introduction to Statistical Learning: With Applications in R, edited by G. James , D. Witten , T. Hastie , and R. Tibshirani , 265–301. Springer. 10.1007/978-1-4614-7138-7_7.

[ece373881-bib-0034] Jasinska, A. J. , A. Haghani , J. A. Zoller , et al. 2021. “Epigenetic Clock and Methylation Studies in Vervet Monkeys.” GeroScience 44, no. 2: 699–717. 10.1007/s11357-021-00466-3. 34591235 PMC9135907

[ece373881-bib-0035] Jones, M. J. , S. J. Goodman , and M. S. Kobor . 2015. “DNA Methylation and Healthy Human Aging.” Aging Cell 14, no. 6: 924–932. 10.1111/acel.12349. 25913071 PMC4693469

[ece373881-bib-0036] Kampkötter, A. , C. Timpel , R. F. Zurawski , et al. 2008. “Increase of Stress Resistance and Lifespan of *Caenorhabditis elegans* by Quercetin.” Comparative Biochemistry and Physiology Part B: Biochemistry and Molecular Biology 149, no. 2: 314–323. 10.1016/j.cbpb.2007.10.004. 18024103

[ece373881-bib-0037] Kelley, J. L. , M.‐C. Yee , A. P. Brown , et al. 2016. “The Genome of the Self‐Fertilizing Mangrove Rivulus Fish, *Kryptolebias marmoratus*: A Model for Studying Phenotypic Plasticity and Adaptations to Extreme Environments.” Genome Biology and Evolution 8, no. 7: 2145–2154. 10.1093/gbe/evw145. 27324916 PMC4987111

[ece373881-bib-0038] Kim, B.‐H. , K. Nho , Y.‐N. Huang , et al. 2023. “Genome‐Wide Association Meta‐Analysis Identifies a Novel LRBA Locus for Brain Age Acceleration in Two Independent Korean Cohorts.” Alzheimer's & Dementia 19, no. S24: e082848. 10.1002/alz.082848.

[ece373881-bib-0039] Konstantinidis, I. , P. Sætrom , and J. M. O. Fernandes . 2023. “Genome‐Wide Hydroxymethylation Profiles in Liver of Female Nile Tilapia With Distinct Growth Performance.” Scientific Data 10, no. 1: 114. 10.1038/s41597-023-01996-5. 36859394 PMC9977925

[ece373881-bib-0040] Kuhn, M. , and K. Johnson . 2013. Applied Predictive Modeling. Springer. 10.1007/978-1-4614-6849-3.

[ece373881-bib-0041] Levine, M. E. , A. T. Lu , A. Quach , et al. 2018. “An Epigenetic Biomarker of Aging for Lifespan and Healthspan.” Aging (Albany NY) 10, no. 4: 573–591. 10.18632/aging.101414. 29676998 PMC5940111

[ece373881-bib-0042] Lin, F. , and H. J. Worman . 1993. “Structural Organization of the Human Gene Encoding Nuclear Lamin A and Nuclear Lamin C.” Journal of Biological Chemistry 268, no. 22: 16321–16326. 10.1016/S0021-9258(19)85424-8. 8344919

[ece373881-bib-0043] Lister, R. , E. A. Mukamel , J. R. Nery , et al. 2013. “Global Epigenomic Reconfiguration During Mammalian Brain Development.” Science 341, no. 6146: 1237905. 10.1126/science.1237905. 23828890 PMC3785061

[ece373881-bib-0044] Liu, L. , T. van Groen , I. Kadish , and T. O. Tollefsbol . 2009. “DNA Methylation Impacts on Learning and Memory in Aging.” Neurobiology of Aging 30, no. 4: 549–560. 10.1016/j.neurobiolaging.2007.07.020. 17850924 PMC2656583

[ece373881-bib-0045] López‐Otín, C. , M. A. Blasco , L. Partridge , M. Serrano , and G. Kroemer . 2013. “The Hallmarks of Aging.” Cell 153, no. 6: 1194–1217. 10.1016/j.cell.2013.05.039. 23746838 PMC3836174

[ece373881-bib-0046] López‐Otín, C. , M. A. Blasco , L. Partridge , M. Serrano , and G. Kroemer . 2023. “Hallmarks of Aging: An Expanding Universe.” Cell 186, no. 2: 243–278. 10.1016/j.cell.2022.11.001. 36599349

[ece373881-bib-0047] Lu, A. T. , Z. Fei , A. Haghani , et al. 2023. “Universal DNA Methylation Age Across Mammalian Tissues.” Nature Aging 3, no. 9: 1144–1166. 10.1038/s43587-023-00462-6. 37563227 PMC10501909

[ece373881-bib-0048] Mayne, B. , O. Berry , and S. Jarman . 2021. “Optimal Sample Size for Calibrating DNA Methylation Age Estimators.” Molecular Ecology Resources 21, no. 7: 7. 10.1111/1755-0998.13437. PMC8518423 34053192

[ece373881-bib-0049] Meyer, D. H. , and B. Schumacher . 2024. “Aging Clocks Based on Accumulating Stochastic Variation.” Nature Aging 4, no. 6: 871–885. 10.1038/s43587-024-00619-x. 38724736 PMC11186771

[ece373881-bib-0050] Moqri, M. , C. Herzog , J. R. Poganik , et al. 2023. “Biomarkers of Aging for the Identification and Evaluation of Longevity Interventions.” Cell 186, no. 18: 3758–3775. 10.1016/j.cell.2023.08.003. 37657418 PMC11088934

[ece373881-bib-0084] Newediuk, L. , E. S. Richardson , A. M. Bohart , A. Roberto‐Charron , C. J. Garroway , and M. J. Jones . 2025. “Designing Epigenetic Clocks for Wildlife Research.” Molecular Ecology Resources 25, no. 7: e14120. 10.1111/1755-0998.14120. 40326643 PMC12415925

[ece373881-bib-0051] Nilsen, F. M. , B. B. Parrott , J. A. Bowden , et al. 2016. “Global DNA Methylation Loss Associated With Mercury Contamination and Aging in the American Alligator ( *Alligator mississippiensis* ).” Science of the Total Environment 545–546: 389–397. 10.1016/j.scitotenv.2015.12.059. PMC4972023 26748003

[ece373881-bib-0052] Pellegrini, C. , C. Pirazzini , C. Sala , et al. 2021. “A Meta‐Analysis of Brain DNA Methylation Across Sex, Age, and Alzheimer's Disease Points for Accelerated Epigenetic Aging in Neurodegeneration.” Frontiers in Aging Neuroscience 13: 639428. 10.3389/fnagi.2021.639428. 33790779 PMC8006465

[ece373881-bib-0053] Pietsch, K. , N. Saul , R. Menzel , S. R. Stürzenbaum , and C. E. W. Steinberg . 2009. “Quercetin Mediated Lifespan Extension in *Caenorhabditis elegans* Is Modulated by Age‐1, Daf‐2, Sek‐1 and Unc‐43.” Biogerontology 10, no. 5: 565–578. 10.1007/s10522-008-9199-6. 19043800

[ece373881-bib-0054] Piferrer, F. , and D. Anastasiadi . 2023. “Age Estimation in Fishes Using Epigenetic Clocks: Applications to Fisheries Management and Conservation Biology.” Frontiers in Marine Science 10: 2–5. 10.3389/fmars.2023.1062151.

[ece373881-bib-0055] Piferrer, F. , E. A. Miska , and D. Anastasiadi . 2024. “Chapter 10—Epigenetics in Fish Evolution.” In On Epigenetics and Evolution, edited by C. M. Guerrero‐Bosagna , 283–306. Academic Press. 10.1016/B978-0-443-19051-3.00010-3.

[ece373881-bib-0056] Raddatz, G. , S. Hagemann , D. Aran , et al. 2013. “Aging Is Associated With Highly Defined Epigenetic Changes in the Human Epidermis.” Epigenetics & Chromatin 6, no. 1: 36. 10.1186/1756-8935-6-36. 24279375 PMC3819645

[ece373881-bib-0058] Ruetz, T. J. , A. N. Pogson , C. M. Kashiwagi , et al. 2024. “CRISPR–Cas9 Screens Reveal Regulators of Ageing in Neural Stem Cells.” Nature 634, no. 8036: 1150–1159. 10.1038/s41586-024-07972-2. 39358505 PMC11525198

[ece373881-bib-0059] Salminen, A. 2022. “Aryl Hydrocarbon Receptor (AhR) Reveals Evidence of Antagonistic Pleiotropy in the Regulation of the Aging Process.” Cellular and Molecular Life Sciences 79, no. 9: 489. 10.1007/s00018-022-04520-x. 35987825 PMC9392714

[ece373881-bib-0060] Seale, K. , S. Horvath , A. Teschendorff , N. Eynon , and S. Voisin . 2022. “Making Sense of the Ageing Methylome.” Nature Reviews Genetics 23, no. 10: 585–605. 10.1038/s41576-022-00477-6. 35501397

[ece373881-bib-0061] Serna, E. , D. Verdú , A. Valls , et al. 2024. “Involvement of Aryl Hydrocarbon Receptor in Longevity and Healthspan: Insights From Humans, Mice, and *C. elegans* .” International Journal of Molecular Sciences 25, no. 18: 9943. 10.3390/ijms25189943. 39337431 PMC11432571

[ece373881-bib-0062] Shastri, G. G. , G. Sudre , K. Ahn , et al. 2024. “Cortico‐Striatal Differences in the Epigenome in Attention‐Deficit/ Hyperactivity Disorder.” Translational Psychiatry 14, no. 1: 189. 10.1038/s41398-024-02896-x. 38605038 PMC11009227

[ece373881-bib-0063] Singhal, R. P. , L. L. Mays‐Hoopes , and G. L. Eichhorn . 1987. “DNA Methylation in Aging of Mice.” Mechanisms of Ageing and Development 41, no. 3: 199–210. 10.1016/0047-6374(87)90040-6. 3431172

[ece373881-bib-0064] Spiers, H. , E. Hannon , S. Wells , B. Williams , C. Fernandes , and J. Mill . 2016. “Age‐Associated Changes in DNA Methylation Across Multiple Tissues in an Inbred Mouse Model.” Mechanisms of Ageing and Development 154: 20–23. 10.1016/j.mad.2016.02.001. 26861500 PMC4798846

[ece373881-bib-0065] Strand, E. L. , R. McBride , E. Robillard , A. G. Bodnar , S. A. Wanamaker , and T. P. O'Donnell . 2025. “Building an ‘Epigenetic Clock’: Utilizing Whole Genome DNA Methylation Patterns to Predict Age in Haddock, *Melanogrammus aeglefinus*.” *bioRxiv*. 10.1101/2025.10.16.682892.

[ece373881-bib-0066] Stubbs, T. M. , M. J. Bonder , A.‐K. Stark , et al. 2017. “Multi‐Tissue DNA Methylation Age Predictor in Mouse.” Genome Biology 18, no. 1: 68. 10.1186/s13059-017-1203-5. 28399939 PMC5389178

[ece373881-bib-0067] Su, H. , F. Li , M. J. Ranek , N. Wei , and X. Wang . 2011. “COP9 Signalosome Regulates Autophagosome Maturation.” Circulation 124, no. 19: 2117–2128. 10.1161/CIRCULATIONAHA.111.048934. 21986281 PMC3211066

[ece373881-bib-0068] Su, H. , J. Li , H. Osinska , et al. 2013. “The COP9 Signalosome Is Required for Autophagy, Proteasome‐Mediated Proteolysis, and Cardiomyocyte Survival in Adult Mice.” Circulation: Heart Failure 6, no. 5: 1049–1057. 10.1161/CIRCHEARTFAILURE.113.000338. 23873473 PMC3835345

[ece373881-bib-0069] Tarkhov, A. E. , T. Lindstrom‐Vautrin , S. Zhang , et al. 2024. “Nature of Epigenetic Aging From a Single‐Cell Perspective.” Nature Aging 4, no. 6: 854–870. 10.1038/s43587-024-00616-0. 38724733

[ece373881-bib-0070] Taylor, D. S. 2000. “Biology and Ecology of *Rivulus marmoratus* : New Insights and a Review.” Florida Scientist 63, no. 4: 4.

[ece373881-bib-0071] Taylor, D. S. 2012. “Twenty‐Four Years in the Mud: What Have we Learned About the Natural History and Ecology of the Mangrove Rivulus, *Kryptolebias marmoratus*?” Integrative and Comparative Biology 52, no. 6: 724–736. 10.1093/icb/ics062. 22576816 PMC3501094

[ece373881-bib-0085] Theng, D. , K. K. Bhoyar , and P. Pawade . 2025. “Feature Selection and Stability Analysis using Ensemble Techniques.” Journal of Intelligent Systems and Internet of Things 16: 41–48. 10.54216/JISIoT.160104.

[ece373881-bib-0072] Thompson, M. J. , B. vonHoldt , S. Horvath , and M. Pellegrini . 2017. “An Epigenetic Aging Clock for Dogs and Wolves.” Aging 9, no. 3: 1055–1068. 10.18632/aging.101211. 28373601 PMC5391218

[ece373881-bib-0073] Thrush, K. L. , D. A. Bennett , C. Gaiteri , et al. 2022. “Aging the Brain: Multi‐Region Methylation Principal Component Based Clock in the Context of Alzheimer's Disease.” Aging 14, no. 14: 5641–5668. 10.18632/aging.204196. 35907208 PMC9365556

[ece373881-bib-0074] Tian, J. , K. Jia , T. Wang , et al. 2024. “Hippocampal Transcriptome‐Wide Association Study and Pathway Analysis of Mitochondrial Solute Carriers in Alzheimer's Disease.” Translational Psychiatry 14, no. 1: 1–15. 10.1038/s41398-024-02958-0. 38858380 PMC11164935

[ece373881-bib-0075] Tong, H. , V. B. Dwaraka , Q. Chen , et al. 2024. “Quantifying the Stochastic Component of Epigenetic Aging.” Nature Aging 4, no. 6: 886–901. 10.1038/s43587-024-00600-8. 38724732 PMC11186785

[ece373881-bib-0076] Unnikrishnan, A. , N. Hadad , D. R. Masser , J. Jackson , W. M. Freeman , and A. Richardson . 2018. “Revisiting the Genomic Hypomethylation Hypothesis of Aging.” Annals of the New York Academy of Sciences 1418, no. 1: 69–79. 10.1111/nyas.13533. 29363785 PMC5934293

[ece373881-bib-0078] Weber, D. N. , A. T. Fields , W. F. Patterson , B. K. Barnett , C. M. Hollenbeck , and D. S. Portnoy . 2022. “Novel Epigenetic Age Estimation in Wild‐Caught Gulf of Mexico Reef Fishes.” Canadian Journal of Fisheries and Aquatic Sciences 79, no. 1: 1–5. 10.1139/cjfas-2021-0240.

[ece373881-bib-0077] Weber, D. , J. T. Wyffels , C. Buckner , et al. 2024. “Noninvasive, Epigenetic Age Estimation in an Elasmobranch, the Cownose Ray ( *Rhinoptera bonasus* ).” Scientific Reports 14, no. 1: 26261. 10.1038/s41598-024-78004-2. 39482525 PMC11528000

[ece373881-bib-0079] Wei, N. , and X. W. Deng . 2003. “The COP9 Signalosome.” Annual Review of Cell and Developmental Biology 19: 261–286. 10.1146/annurev.cellbio.19.111301.112449. 14570571

[ece373881-bib-0080] Wilson, V. L. , R. A. Smith , S. Ma , and R. G. Cutler . 1987. “Genomic 5‐Methyldeoxycytidine Decreases With Age.” Journal of Biological Chemistry 262, no. 21: 9948–9951. 10.1016/S0021-9258(18)61057-9. 3611071

[ece373881-bib-0081] Yang, J. , L. Yu , C. Gaiteri , et al. 2015. “Association of DNA Methylation in the Brain With Age in Older Persons Is Confounded by Common Neuropathologies.” International Journal of Biochemistry & Cell Biology 67: 58–64. 10.1016/j.biocel.2015.05.009. 26003740 PMC4564337

[ece373881-bib-0082] Zoller, J. A. , A. T. Lu , A. Haghani , S. Horvath , and T. Robeck . 2025. “Enhancing Epigenetic Aging Clocks in Cetaceans: Accurate Age Estimations in Small Endangered Delphinids, Killer Whales, Pilot Whales, Belugas, Humpbacks, and Bowhead Whales.” Scientific Reports 15, no. 1: 4048. 10.1038/s41598-025-86705-5. 39900928 PMC11791194

[ece373881-bib-0083] Zou, C. , L. Su , M. Pan , et al. 2023. “Exploration of Novel Biomarkers in Alzheimer's Disease Based on Four Diagnostic Models.” Frontiers in Aging Neuroscience 15: 1079433. 10.3389/fnagi.2023.1079433. 36875704 PMC9978156

