# Supplementary figures and images for: Epigenetic Aging in Brain Tissue of the Self‐Fertilizing Vertebrate, Kryptolebias marmoratus
Source: Ecol Evol. 2026 Jun 21;16(6):e73881. doi: 10.1002/ece3.73881 (PMC13283775; doi:10.1002/ece3.73881)

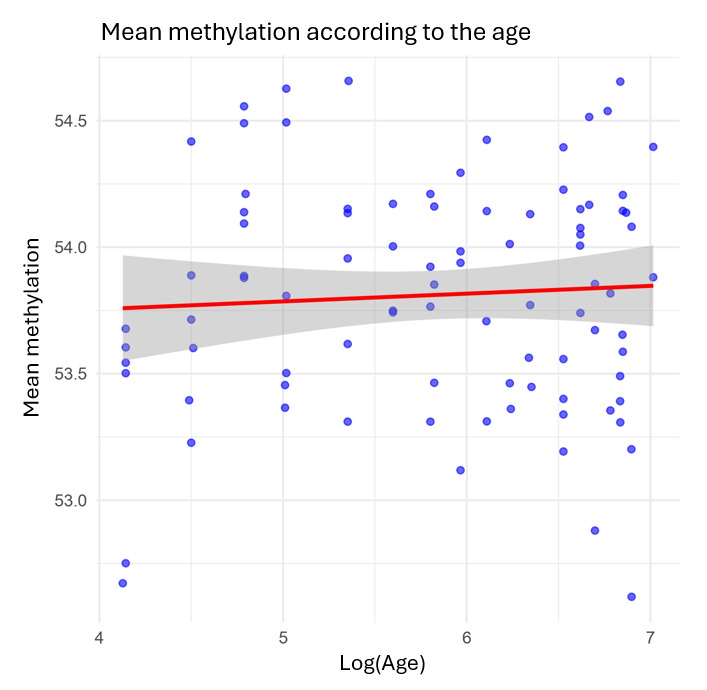

Supplement: Supplementary file 1 — Figure S1: Global methylation level with age. [file ECE3-16-e73881-s001.jpg]

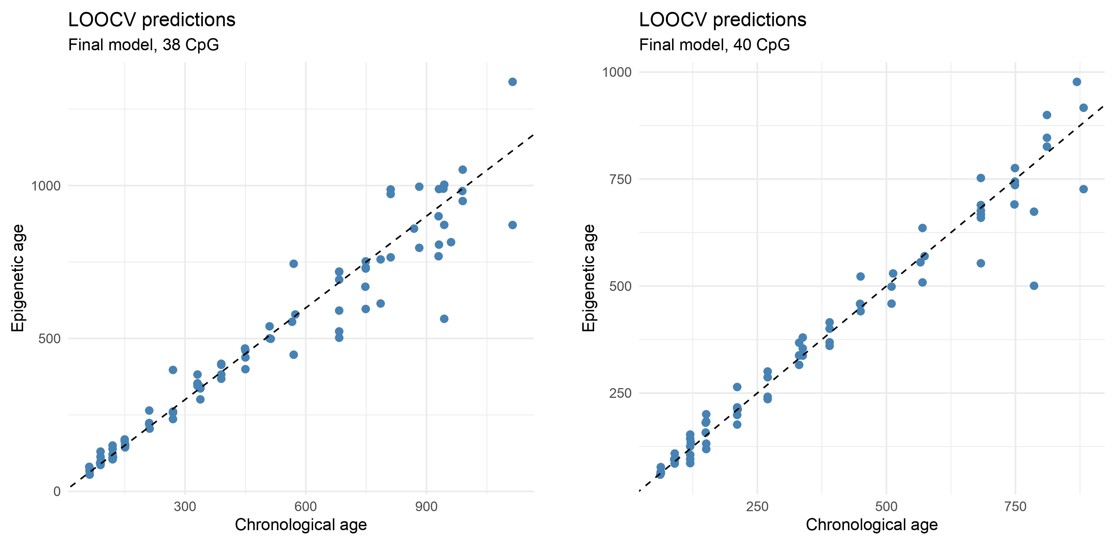

Supplement: Supplementary file 2 — Figure S2: Leave‐one‐out cross validation for both final models (all samples included on the left, only samples < 900 days on the right). [file ECE3-16-e73881-s003.jpg]

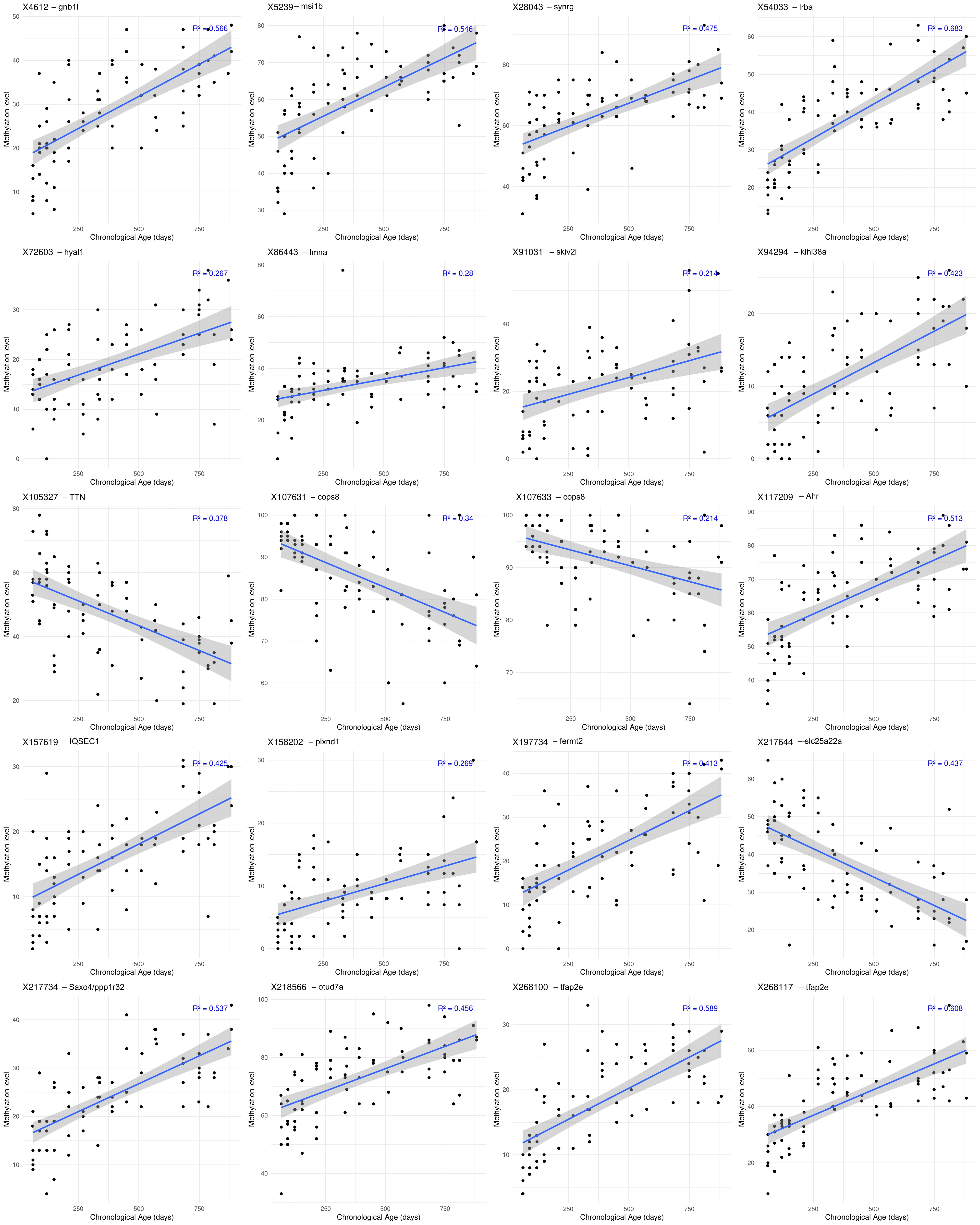

Supplement: Supplementary file 3 — Figure S3: DNA methylation level of 40 selected CpG sites across chronological age. [file ECE3-16-e73881-s004.zip › S3a.jpg]

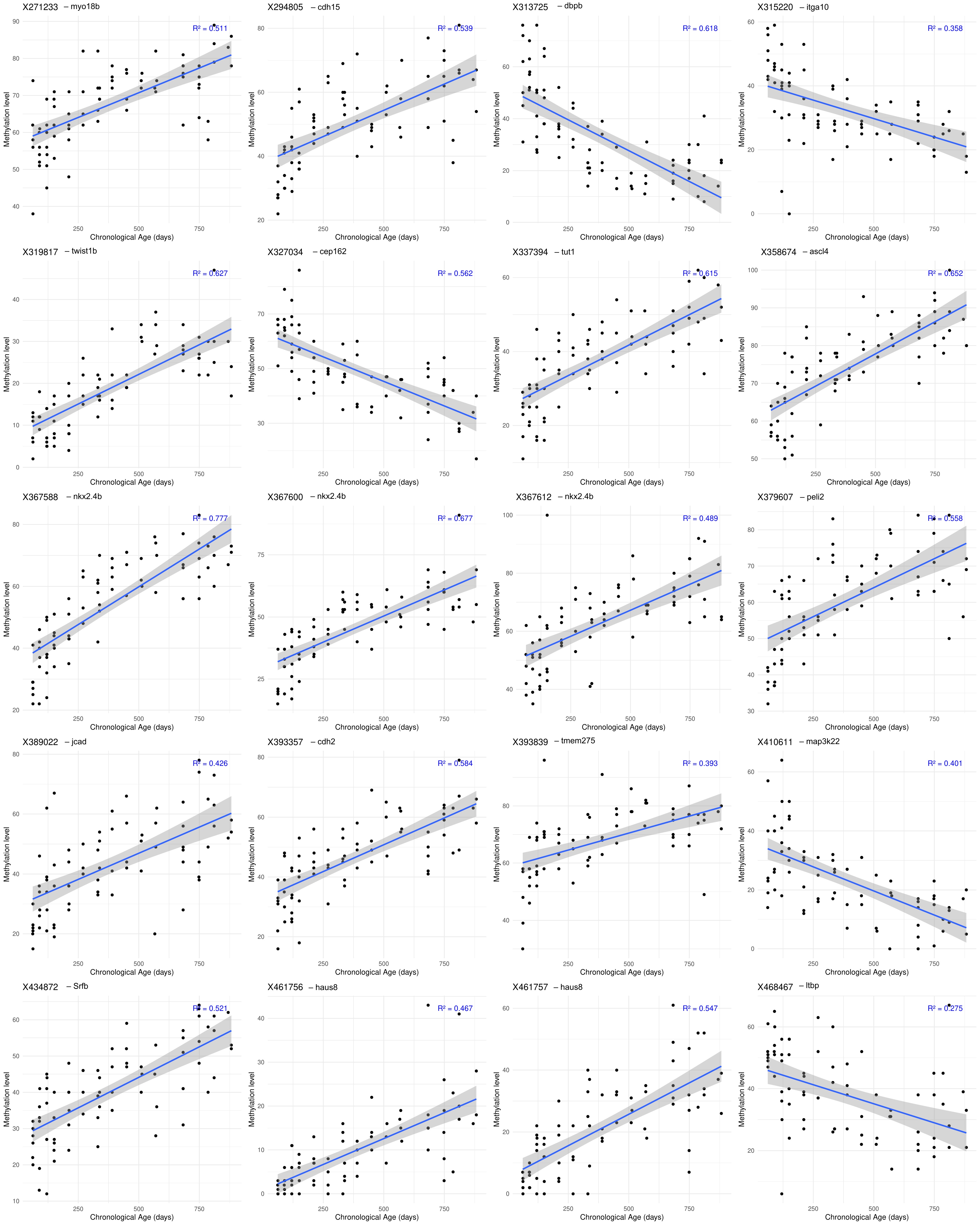

Supplement: Supplementary file 3 — Figure S3: DNA methylation level of 40 selected CpG sites across chronological age. [file ECE3-16-e73881-s004.zip › S3b.jpg]
